# Watershed characteristics shape the landscape genetics of brook stickleback (*Culaea inconstans*) in shallow prairie lakes

**DOI:** 10.1002/ece3.2885

**Published:** 2017-03-28

**Authors:** Cory S. Kremer, Steven M. Vamosi, Sean M. Rogers

**Affiliations:** ^1^ Department of Biological SciencesUniversity of CalgaryCalgaryABCanada

**Keywords:** bottlenecks, *Culaea inconstans*, fragmentation, landscape genetics, winterkill

## Abstract

Investigating the consequences of landscape features on population genetic patterns is increasingly important to elucidate the ecological factors governing connectivity between populations and predicting the evolutionary consequences of landscapes. Small prairie lakes in Alberta, Canada, and the brook stickleback (*Culaea inconstans*) that inhabit them, provide a unique aquatic system whereby populations are highly isolated from one another. These heterogeneous and extreme environments are prone to winterkills, an event whereby most of the fish die and frequent bottlenecks occur. In this study, we characterized the genetic population structure of brook stickleback among several lakes, finding that the species is hierarchically influenced by within‐lake characteristics in small‐scale watersheds. Landscape genetic analyses of the role of spatial features found support for basin characteristics associated with genetic diversity and bottlenecks in 20% of the sampled lakes. These results suggest that brook stickleback population genetic patterns may be driven, at least in part, by ecological processes that accelerate genetic drift and landscape patterns associated with reduced dispersal. Collectively, these results reinforce the potential importance of connectivity in the maintenance of genetic diversity, especially in fragmented landscapes.

## 1 Introduction

Elucidating how landscape features may influence genetic drift, gene flow, and selection in natural systems remains a significant challenge in ecology and evolution (Manel, Schwartz, Luikart, & Taberlet, 2003; Sork & Waits, 2010; Storfer et al., 2007). This challenge lies principally in the ability to disentangle the relative roles of gene flow and genetic drift compared to selective responses in association with heterogeneous landscapes (Holderegger & Wagner, 2006). Landscape genetic approaches have primarily focused on how landscape features influence gene flow because of gene flow's homogenizing influence on population genetic diversity (Storfer, Murphy, Spear, Holderegger, & Waits, 2010), reducing the propensity for local adaptation (Kawecki & Ebert, 2004). However, in fragmented landscapes, lower gene flow may lead to more pronounced local regimes of selection and drift, characteristic of the genetic diversity found in such isolated patches (e.g., Keller & Largiadèr, 2003). Many such barriers to gene flow in fragmented landscapes have now been identified in a multitude of systems (e.g., Funk et al., 2005; Keyghobadi, Roland, & Strobeck, 1999; Proctor, Mclellan, Strobeck, & Barclay, 2004). Nonetheless, the effects of habitat fragmentation on species remain mixed, with some exhibiting significant changes in genetic diversity while others are seemingly unaffected (Storfer et al., 2010), reinforcing the need for species‐specific work to characterize generalities about the influence of landscape variables on population genetic diversity.

Freshwater systems provide a unique opportunity to investigate the population genetic consequences of fragmented landscapes, because streams and lakes may often impose limitations to dispersal between populations (e.g., Bahr & Shrimpton, 2004; Petty, Lamothe, & Mazik, 2005). For example, genetic population structure patterns of freshwater fish populations may reflect the corresponding hydrological structures where such species are found (e.g., Castric, Bonney, & Bernatchez, 2001; Huey, Baker, & Hughes, 2008; Meeuwig, Guy, Kalinowski, & Fredenberg, 2010). In other cases, freshwater species that are not limited by hydrology (e.g., due to terrestrial stages of their life histories) exhibit contemporary gene flow patterns associated with how organisms use landscapes (e.g., Chaput‐Bardy, Lemaire, Picard, & Secondi, 2008; Finn & Adler, 2006).

Three nonmutually exclusive hypotheses have been proposed with respect to the population genetic patterns predicted for aquatic organisms inhabiting stream networks as alternatives to panmixia (Hughes, Schmidt, & Finn, 2009). The “Death Valley Model” (DVM; Meffe & Vrijenhoek, 1988), modeled after pupfishes and killifishes of California's Death Valley, predicts that divergence will occur among isolated stream networks that do not have immediate hydrological connections, resulting in an imbalance between drift and gene flow (where gene flow is effectively zero). In these cases, populations are allopatric and inevitably expected to diverge because no forces (other than selection) exist to prevent differentiation. The “Stream Hierarchy Model” (SHM; Meffe & Vrijenhoek, 1988) predicts that the degrees of connectivity and gene flow will vary depending on the spacing of populations within the hierarchical network structure in dendritic stream networks (Hughes et al., 2009). In these cases, genetic variance associated with population structure is expected to partition significantly among drainage basins at any spatial scale at which basins can be defined within the stream network (Hughes et al. 2007). The “Headwater Model” (HM; Hughes et al., 2009) proposes that species inhabiting lower elevation streams should experience higher rates of gene flow because the habitats that they inhabit are more connected as streams join together at lower elevations, and share environments that such species are adapted to. All of these models predict that the hydrological structure of landscapes should influence gene flow in aquatic organisms. Yet, whether genetic diversity within populations is the result of between‐lake processes (i.e., landscapes and the propensity of lakes and surrounding watershed to facilitate dispersal) versus within‐lake processes (i.e., environmental factors that influence demographic processes) remains unknown.

The shallow lakes of the northwestern plains of North America comprise landscape features that facilitate testing these competing hypotheses. Aquatic organisms are hierarchically distributed in relatively discrete units (e.g., individuals found within a single lake) within watersheds in this environment. Lakes are intermittently connected by streams and also prone to winter anoxia resulting from ice cover (Barica & Mathias, 1979), potentially leading to “winterkills” of fish populations (Robinson & Tonn, 1989; Tonn & Magnuson, 1982). For example, fathead minnow (Pimephales promelas) populations can be reduced by as much as 90% during an anoxic year (Danylchuck & Tonn, 2003). Associations between physical characteristics of basins (e.g., depth, surface area; Meding & Jackson, 2003) and anoxic conditions, and between anoxic conditions and high population size variability and species assemblage (Robinson & Tonn, 1989), suggest that fish populations inhabiting these lakes should be indirectly affected by basin landscape characteristics as a result of processes such as anoxia. Brook stickleback (Culaea inconstans) is a hypoxia‐tolerant, forage fish species found in many of these basins where they are typically found in small lotic and lentic systems (Robinson & Tonn, 1989). Brook sticklebacks are known to travel up runoff during the spring or following heavy precipitation events (Nelson & Paetz, 1992), indicating that their propensity for dispersal may be high. Spawning occurs in late spring and early summer: when males secure and defend a territory, construct a nest out of vegetation, and attract gravid females (Nelson & Paetz, 1992). Brook stickleback life spans are generally thought to be short, typically spanning <2 years (King & Cone, 2008). Brook stickleback exhibits behavioral (Magnuson, Beckel, Mills, & Brandt, 1985) and physiological adaptations (Klinger, Magnuson, & Gallepp, 1982) to anoxic conditions and are often found where other fish species are unable to persist (Nelson & Paetz, 1992; Robinson & Tonn, 1989). The characteristics of brook stickleback and the systems that they are found in provided an excellent system to study the population genetic consequences of habitat fragmentation and variability.

In this study we characterized the genetic population structure of brook stickleback inhabiting a series of shallow lakes in the northwestern prairies of Alberta to test predictions associated with the three predominant hypotheses for the role of fragmentation in aquatic environments. We predicted that populations of brook stickleback should exhibit patterns of genetic diversity characteristic of recent population bottlenecks as a result of winterkills. We measured 10 landscape metrics and, using an information‐theoretic approach, tested the competing hypotheses that genetic diversity within populations would either be the result of between‐lake processes (i.e., the propensity of a lake and its surroundings to facilitate migration and movement controls genetic diversity) versus within‐lake processes (i.e., features of lakes influence the demographic processes that occur within a lake, and these influence genetic diversity through effective population size and drift). This approach permitted testing these landscape genetic consequences in brook stickleback inhabiting fragmented and heterogeneous environments.

## 2 Methods

### 2.1 Field collections

**Figure 1:**
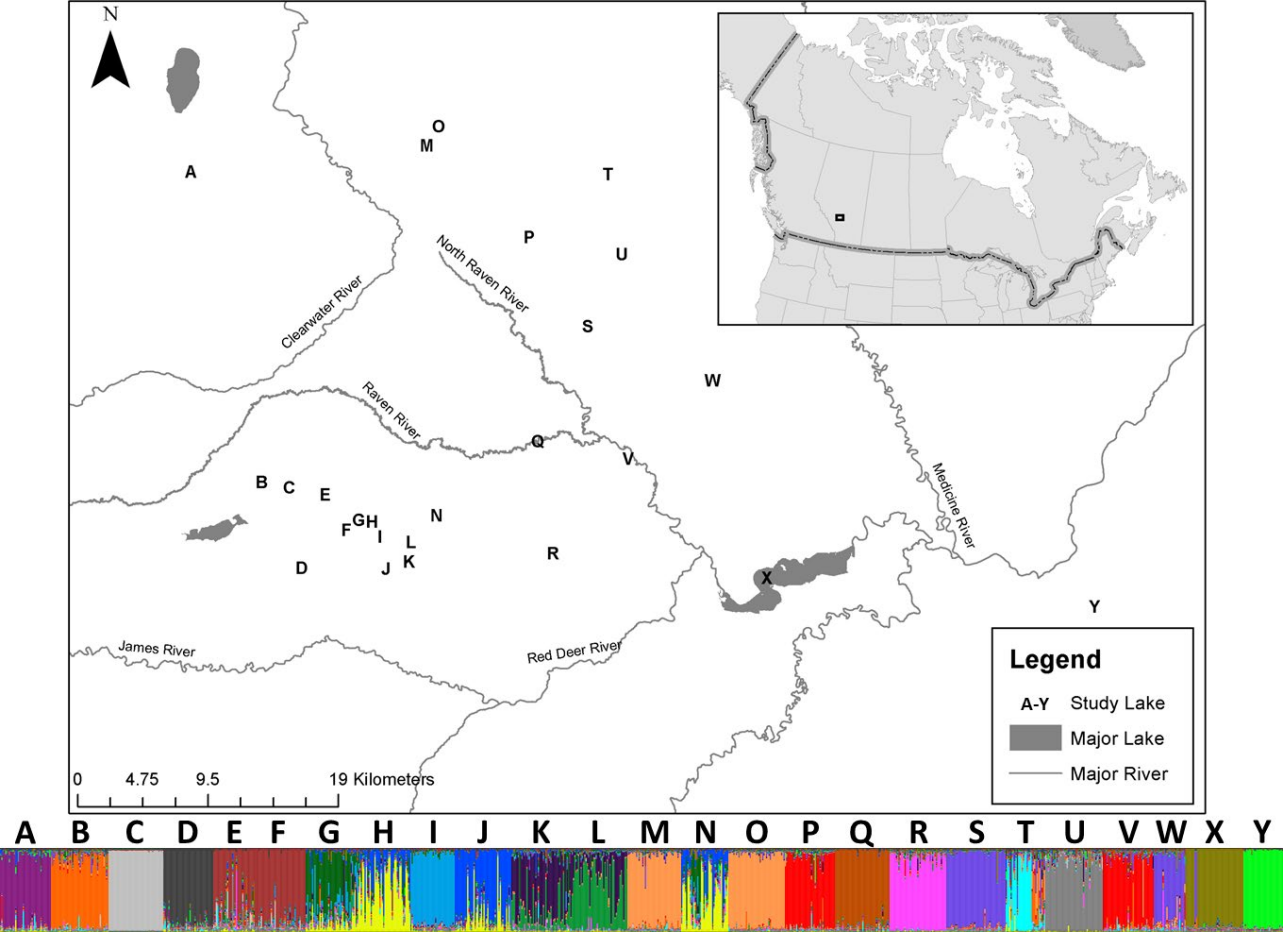
A map of the lakes sampled for brook stickleback in small Central Alberta lakes with the average population STRUCTURE cluster assignment overlaid on top of the location that the population was collected

Baited Gee minnow traps were set in 52 lakes near Caroline, Alberta, during the summers of 2011 and 2012 (Figure 1; Table S1). Sites were selected to represent a range of pairwise Euclidean distances and hydrological distances in addition to sampling multiple lakes within and between watersheds. Brook stickleback (*N* = up to 48 per site) were haphazardly selected from the captured group and euthanized using an overdose of eugenol in accordance with the Canadian Council for Animal Care before being preserved in 95% ethanol for subsequent DNA isolation. All samples were collected under our fish research license issued by Alberta Environment Sustainable Resource and Development Fish and Wildlife, while all methods were approved by the University of Calgary's Life and Environmental Sciences Animal Care Committee.

### 2.2 Environmental variables

To facilitate tests of the effects of environmental variables on genetic diversity, we generated 10 landscape metrics using a variety of data sources (Table 1). Geographic vector data were acquired from the AltaLIS Ltd database, and a 1:20,000 digital elevation model was acquired from the GEODE consortium. All GIS operations and layer modifications were conducted using ArcGIS 10.0 (ERSI). The layers acquired from these data sources were then used to calculate the landscape metrics in conjunction with depth measurements collected from the field, and Alberta Environment Sustainable Resource and Development data when available.

**Table 1:** Linear model classification and justification for describing allelic and private allelic richness

Hypothesis	Subhypothesis	Model	*K*	Ecological justification
Between‐Lake Processes	Hydrological Migration	Connected	2	Corridors are an important feature for habitat connectivity (e.g., Hass 1995). Stream connections to a lake may provide a point of entry for migrants to a given lake.
		Flow	2	Flow accumulation is a metric of the amount of water that flows into a given lake, and the number of migrants into a lake may be associated with the amount of water which travels through it. Furthermore, lakes with little flow are at the high points within drainages, and thus have less opportunity to exchange migrants through hydrological connections (see Hughes et al., 2009).
		Connected*Flow	4	The number of migrants entering a lake may be a function of not only the flow accumulation that a lake experiences, but also weather that flow is connected to an inflow or an outflow.
		Connected*Flow*Elevation	8	Lake connectivity (connected*flow) may be influenced by elevation, whereby lakes at high elevation are more difficult to travel to as a result of the potential vertical distance that migrants may need to cover.
	Euclidian migration	NearLakes	2	Patches in close proximity to other patches may provide stepping stones for migrants or directly provide migrants, increasing or decreasing genetic diversity depending on the system (e.g., Young et al. 1996).
		Slope	2	Lakes surrounded by a sloped environment may be difficult to enter for potential migrants (e.g., Low et al. 2006). In the case of the brook stickleback flooding events in lakes which are surrounded by steep slope may impede the ability of migrants to migrate into a lake of interest, and reduce the genetic diversity added to a lake via gene flow.
		Slope + NearLakes	3	Lakes which have a high propensity to act as stepping stones to one another may have migration rates influenced as a result of topographic complexity surrounding a given lake.
	Mixed migration	Connected*Flow + Slope + NearLakes	6	Both the Euclidean and Hydrological Migration Hypotheses may describe genetic diversity.
Within‐lake processes	Drift/Demographic	Area	2	Larger lakes are associated with larger populations, and with larger populations drift and the associated stochastic loss of alleles becomes less prominent.
		Depth	2	Shallow lakes have been associated with winter hypoxia (Barica & Mathias, 1979). Deeper lakes may experience less hypoxia and so populations in deeper lakes experience less demographic stochasticity and be less influenced by drift (more genetic diversity).
		Radiation	2	Increased direct solar radiation slow the rate of freezing of lakes during the winter and increase the rate of thaw during spring, leading to shorter periods of ice cover (a variable linked to winter hypoxia; Meding & Jackson, 2001).
		Reoccur	2	Variability of a lakes shape and size during its history could correlate with the demographic fluctuations in the lake's past, and thus influenced the importance of genetic drift in the lake's past, influencing genetic diversity at the time of sampling. Historical processes are known to have a profound impact on current population genetic patterns in several aquatic species (e.g., Jocelyn et al. 2005).
		Shape	2	The shape of a lake may influence the habitat complexity (more littoral zone, possible refugia during winter), and influence the amount of functional habitat available (see Dolson et al. 2009). Brook stickleback are known to use oxygen bubbles trapped under ice as an oxygen source during winter (Klinger et al., 1982). Lake shape could be indicative of heterogeneity, and the propensity of a lake to form oxygen bubbles.
		Area*Depth	4	Area and depth may interact to influence the demographic stability of a resident population, allowing for larger or more stable populations, reducing the influence of drift. Area and depth interactions may also provide a metrics insights into a lakes volume.
		Area*Depth + Shape + Radiation + Reoccur	7	All lake metrics have an influence on how the size and/or the demographic stability of populations, reducing genetic drift and the associated loss of alleles.
Mixed processes		Elevation	2	Elevation can be interpreted as important in two distinct ways: (1) Elevation may have an influence on ice thaw dates, and temperature, and (2) elevation may influence the ability of migrants to travel to a given lake. Other studies have identified elevation as an important determinant of genetic diversity in amphibian breeding ponds (Funk et al., 2005).
		Slope + Area + NearLakes*Elevation	6	Genetic diversity is determined by both gene flow and genetic drift. Gene flow may primarily be determined by the Euclidean migration hypothesis, and genetic drift may be primarily controlled by population size which may correlate with patch size.
		Area*Depth + Shape + Slope + NearLakes	7	Genetic diversity is determined by both gene flow and genetic drift. Gene flow may primarily be described by the Euclidean migration hypothesis. Genetic drift is determined by current patch features and processes.
		Area*Depth + Shape + Connected*Flow	8	Genetic diversity is determined by both gene flow and genetic drift. Gene flow may primarily be described by the hydrological migration hypothesis. Genetic drift is determined by current patch features and processes.
Global		All models combined.	17	All models cumulatively described describe genetic diversity.

Base lake features included several polygon categories representing fluvial water bodies of lentic systems (as samples were only collected from lakes) were assimilated into individual features using the *dissolve* tool. Area was hypothesized to influence genetic diversity (e.g., Hanfling and Brandl 1998), with larger lakes potentially containing more brook stickleback. Lake shape was measured using a perimeter squared to area ratio, as shape has been shown to vary with littoral zone size (Dolson et al. 2009), which could be indicative of habitat availability and heterogeneity. Perimeter squared to area ratio was used because it was not correlated with area but could still be interpreted as a lake shape metric. Elevation was calculated by calculating the mean digital elevation model (DEM) value within each sampled lake using the *zonal statistics to table* tool, with genetic diversity having been linked to elevation in frog populations (Funk et al., 2005).

Euclidean distances between all lentic water bodies, distances to the nearest five lentic water bodies, and water body connectivity to stream networks, were calculated using the *near to table* tool in ArcGIS. Lakes that had a nearest stream value of zero (i.e., when steam polylines overlapped with lake polygons) were classified as connected, and those with values greater than zero were classified as unconnected. Hydrological distances between lakes that were connected to a stream network were calculated by first converting the stream network polyline layer into a network dataset using ArcCatalog 10. The closest facility analysis was then used, where lake features were converted to points using the feature to point tool and added to the closest facility analysis using the add locations tool (network analyst). The closest facility tool is typically used to identify the shortest distance following a road network between locations of emergencies (incidents), and the facilities required for assistance for such incidents. All lake points where added both as incidents and as facilities, and the stream network was used as the road network. Using the closest facility analysis, pairwise distances via known lotic pathways were calculated between lakes.

Surrounding topographic complexity was estimated because gene flow may be restricted by slope (Lowe et al. 2006). Surrounding slope was measured by calculating the mean slope within a buffer zone around each sampled lake. A 500 m buffer zone, minimizing overlapping between neighboring lakes, was created around each lake using the buffer tool. Slope for the DEM was calculated using the slope tool. Summary statistics were then calculated for slope within each sample lake's buffer using the zonal statistics to table tool.

A measure of the flow accumulation was used to characterize different watershed scales. The location of a population in a drainage may be a predictor of genetic diversity, as populations found lower in a drainage might be more likely to receive migrants from other populations located in the same (or adjacent) drainages. We used a measure of flow accumulation to estimate drainage and a measure of flow direction to estimate flow accumulation in ArcGIS. We converted these layers to define “pour points” in the watershed delineation according to three different scales of flow accumulation (1%, 0.02%, and 0.01%) representative of large‐, medium‐, and small‐scale watersheds, respectively (Table 2). The memberships of each sampled lake into each watershed scale were then extracted using the zonal statistics to table tool. Stickleback were then grouped for the AMOVA to test population structure at each of these watershed scales.

**Table 2:** AMOVA of brook stickleback populations structured by lake grouping at three different watershed scales (determined by flow accumulation and watershed analysis in ArcGIS—see Methods for details). An asterisk indicates significance following 100,172 permutations and including a sequential Bonferroni multiple test correction

Watershed scale	Image of watersheds (red line = 8 km)	Source of variation	Sum of squares	Variance components	Percentage variation
Large	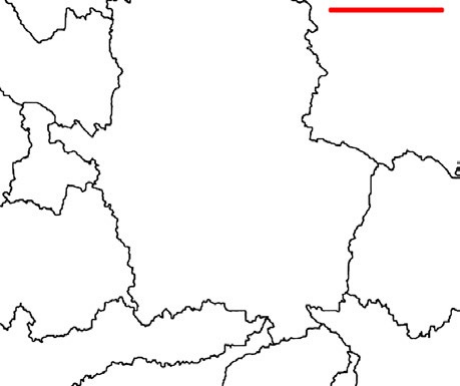	F_CT_	146.735	0.07520	5.30*
		*F*_SC_	319.267	0.23402	16.50*
		*F*_ST_	1823.689	1.10930	78.20*
		Total	2289.691	1.41852	
Medium	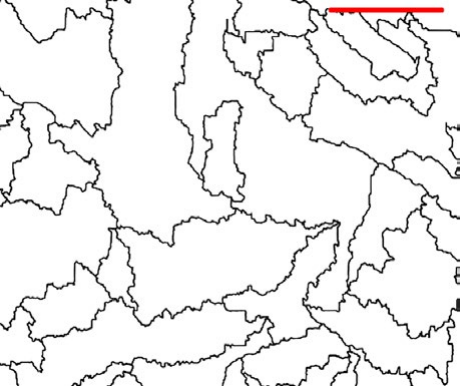	F_CT_	131.233	0.13024	9.68*
		*F*_SC_	160.148	0.17824	13.25*
		*F*_ST_	1156.914	1.03666	77.07*
		Total	1448.296	1.34515	
Small	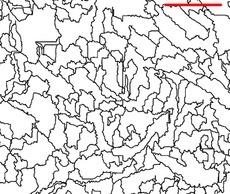	F_CT_	114.154	0.12381	9.45*
		*F*_SC_	119.582	0.14827	11.32*
		*F*_ST_	1059.425	1.03763	79.23*
		Total	1293.161	1.30971	

We generated a measure of solar radiation received by each lake because incoming energy may have a substantial impact on identity and abundance of organisms present (e.g., Nixon 1988, Sterner et al. 1997). As our estimate incorporates topography and given the latitude and how low the sun rises on the horizon at the study location during the winter, solar radiation has the potential to provide some insight on to how surrounding topography could influence population level processes. Direct solar radiation, duration of direct solar radiation, and total solar radiation at the winter solstice were calculated for the points representing the center of 0.001 × 0.001 degree quadrats for all sampled lakes using the points solar radiation tool.

Depths were hypothesized to be predictive of genetic diversity of populations, notably as a factor that influences the decay in oxygen concentration throughout winter (Barica & Mathias, 1979; Meding & Jackson, 2003). Depth data for lakes were generated in one of two ways. When available, provincial government bathymetric maps were used to acquire maximum depths. For lakes lacking these maps, we estimated depths directly using weighted surveying tape at 3–5 sites (depending on the size of the lake and variation in depth measurements) in a uniform distribution across the surface of the lake.

### 2.3 Molecular methods

We characterized 11 polymorphic loci for brook stickleback (Table S2), of which six were originally isolated for threespine stickleback (*Gasterosteus aculeatus*) and five for ninespine stickleback (*Pungitius pungitius*). DNA was isolated using standard phenol–chloroform DNA extraction methods (Sambrook, MacCallum, & Russell, 2001). Reactions were conducted in 10 μl volumes with the following final reaction concentrations: 1× NEB (New England Biolabs) PCR Buffer, 1.92 mmol/L MgSO_4_, 0.5 μmol/L NEB dNTP, 0.5 μmol/L forward primer, 0.5 μmol/L reverse primer, 0.1 μmol/L NEB bovine serum albumin (BSA), 0.03 units/μl NEB TAQ DNA polymerase, and 20 ng of template DNA. Reactions were carried out with the following PCR protocol: 93°C for 180 s, 95°C for 30 s, 56°C for 30 s, 72°C for 30 s, then five cycles of 94°C for 30 s, 56°C for 30 s, 72°C for 30 s, then 35 cycles of 90°C for 30 s, 56°C for 30 s, 72°C for 30 s, and a final extension phase of 72°C for 10 min and 4°C until removal from the thermocycler. Ninespine stickleback primers utilizing an M13 fluorescence tag required the following adjusted reaction conditions of 0.175 μmol/L forward primer and 0.5 μmol/L of the M13 tag, and an additional cycle prior to the 10‐min 72°C stage of the PFC8 PCR protocol, with nine cycles of 94°C for 30 s, 53°C for 45 s, and finally 72°C for 45 s being added. PCR products and alleles were separated and genotyped via electrophoresis on an Applied Biosystems 3500xl sequencer. Alleles were visualized and scored against an internal size standard using GENEMAPPER 4.1 (Applied Biosystems).

### 2.4 Population genetics statistics

A modified version of Fisher's exact test of Hardy–Weinberg proportions was used with 9,999,999 dememorization steps and 9,999,999 Markov chain steps to test for Hardy–Weinberg equilibrium (Guo & Thompson, 1992). Linkage disequilibrium was tested using a likelihood‐ratio test using 99,999 permutations and 20 initial conditions for the expectation‐maximization algorithm (Slatkin & Excoffier, 1996). Both tests were conducted using ARLEQUIN 3.5 (Excoffier & Lischer, 2010). *p*‐values from multiple comparisons from within populations were corrected using a false discovery rate procedure (Benjamini & Yekutieli, 2001) to control for type II errors (Narum, 2006). Following Narum (2006), multiple comparisons were also corrected using the sequential Bonferroni method for *p*‐value corrections for comparison (Holm, 1979).

We employed the algorithm of Dempster, Laird, and Rubin (1977) to estimate null allele frequencies following Chapuis and Estoup (2007) in FREENA, whereby the fit of allele frequency data to Hardy–Weinberg expectations is maximized by varying the estimated frequency of null alleles.

Population structure was assessed using assignment methods implemented in STRUCTURE (Pritchard, Stephens, & Donnelly, 2000), using a 100,000 “burn‐in” period, and 900,000 Monte Carlo Markov Chain steps for one through 25 putative *K* genetic clusters (for each lake sampled). Each STRUCTURE run for each putative population number was replicated 10 times. STRUCTURE provides the natural logarithm of the probability (ln(P)) for each independent run. The runs with the same assumed number of populations (*K*) with the highest ln(P) is regarded as the most likely number of populations. This method has been shown to be particularly sensitive to isolation‐by‐distance patterns (Frantz, Cellina, Krier, Schley, & Burke, 2009; Schwartz & McKelvey, 2009). Therefore, we employed another alternative metric, ∆*K*, to identify the best supported *K* to alleviate STRUCTURE's tendency to overestimate *K* (Evanno, Regnaut, & Goudet, 2005). Both ∆*K* and ln(P) were used to assess population structure.

Individual assignment algorithms such as those in STRUCTURE have stochastic components within them, such that independent trials can produce different results. Furthermore, clusters that are identified by either algorithm are often labeled as different clusters among runs, making it difficult to compare independent runs. We used an algorithm implemented in CLUMPP that maximizes the pairwise similarity between assignments of independent runs of individual assignment algorithms (Jakobsson & Rosenberg, 2007). CLUMPP matches the labels of equivalent clusters between independent individual assignment runs, allowing independent runs to be compared for multimodality (i.e., multiple supported assignment scenarios). We used the “LargeKGreedy” algorithm in CLUMPP to resolve label switching issues that arose from the STRUCTURE individual assignment runs using 1,000,000 permutations and random input orders when a *K* of 15 or more was best supported. If a *K* ≤ 15 was best supported, the “Greedy” algorithm in CLUMPP was used to resolve label switching issues, using 1,000,000 permutations and random input orders. Once clusters labels were adjusted for label switching, they were displayed graphically using DISTRUCT (Rosenberg, 2004).

To test for patterns indicative of historical bottlenecks in each population we used BOTTLENECK 1.2.02 (Cornuet & Luikart, 1996). BOTTLENECK uses the expectation that following a population bottleneck, heterozygosity should be significantly more common than would be expected given the allelic diversity in an assumed mutation–drift equilibrium model for the provided sample. Wilcoxon sign test *p*‐values were estimated using BOTTLENECK with 100,000 replicate estimates of expected heterozygosity under the infinite allele model (IAM, Watterson, 1984), two‐phase model (TPM, Di Rienzo et al., 1994), and the stepwise mutation model (SMM, Chakraborty & Nei, 1977). All three mutation models were tested because there is disagreement in the literature over which model is most relevant to microsatellites (e.g., Neff, Fu, & Gross, 1999), and may produce different outcomes (e.g., Luikart & Cornuet, 1998). If a test of any assumed mutation model resulted in significant excess heterozygosity in the population in question, it was believed to have undergone a putative bottleneck. Populations that had undergone a putative bottleneck were assigned a logit score of one, or zero if they had not, for corresponding logistic regression analyses used to test for a relationship between landscape characteristics and putative bottleneck status.

### 2.5 Landscape genetics statistics

We used an AMOVA (Excoffier, Smouse, & Quattro, 1992) to test for watershed effects at the three different scales of watershed units on genetic differentiation using 99,999 permutations in ARLEQUIN 3.5. As watershed delineation was generated according to three different levels of flow accumulation representative of large‐, medium‐, and small‐scale watersheds (Table 2), hierarchical groups for the AMOVA were determined by genetic memberships to these watersheds, over the three different watershed scales. Significant values resulting from tests of the effect of watershed on different scales were corrected using the sequential Bonferroni correction.

Isolation‐by‐distance and hierarchical structure patterns were assessed using partial Mantel tests, with the responding variable being *F*_ST_/1‐*F*_ST_ (genetic distance, a metric that linearizes *F*_ST_ estimates; Rousset, 1997). *F*_ST_ estimates were corrected for null allele frequencies in FREENA by excluding null allele estimated frequencies (so that allele frequencies for any given locus sums to one minus the estimated frequency of null alleles), which has been demonstrated to provide more accurate *F*_ST_ estimates than uncorrected data (Chapuis & Estoup, 2007). Within the partial Mantels tests that tested for isolation by distance, a pairwise matrix of membership of each population in each cluster (one for shared membership in a cluster, a zero for unshared membership) was used as a covariate, and the measurement of geographic distances was used as the predictor variable. Hierarchical structure was assessed using geographic distance as a covariate and cluster membership as the predictor variable. This partial Mantel approach has been suggested as a means to tease apart hierarchical structure from isolation‐by‐distance patterns (Meirmans, 2012). Where structural algorithms yielded no evidence of substantial structure at a scale larger than an individual population, isolation by distance was tested using a Mantel test without cluster membership as a covariate. Two sets of (partial) Mantel tests were conducted: one on pairwise Euclidean distance between populations, and one conducted on the stream distance between populations where only lakes connected to the main stream network were included. Mantel (or Partial Mantel) Tests were conducted using the “ecodist” R package using 1,000,000 permutations (Goslee & Urban, 2007). Multiple comparisons were accounted for by correcting *p*‐values using the sequential Bonferroni method prior to interpretation.

Landscape influences on genetic diversity were assessed using two metrics of genetic diversity: private allelic richness (number of unique alleles in each population) and allelic richness. These metrics of genetic diversity were used because, for example, heterozygosity decreases at a slower rate than allelic richness during a bottleneck (Nei, Maruyama, & Chakraborty, 1975). Allelic richness indices were controlled for sample size using HP‐RARE that uses rarefaction to control for sample size when estimating for allelic and private allelic richness, correcting for the lowest sample size of 18 (Kalinowski, 2005). We used an information‐theoretic model selection approach to distinguish between two groups of mechanistic hypotheses with regard to how allelic richness and private allelic richness are potentially associated with landscape features. Spatial metrics were accordingly categorized as representing “within‐lake” or “between‐lake” hypotheses (Table 1). Ten univariate models were created for each metric explaining genetic diversity and three multivariate models for each overarching hypothesis (consisting of the variables which had been assigned to each hypothesis). Three “mixed models” were created consisting of variables that originated from both hypotheses. Finally, a “global” model containing all variables was included. Corrected AICc scores (AIC scores corrected for a finite sample size; Burnham and Anderson 2002) of the models were compared. Models within 2.0 AICc units of the “best” model were considered to be equivalent. The simplest model was considered only when a nested model was within 2.0 AICc units.

## 3 Results

A total of 1,123 brook stickleback were genotyped from 25 of 52 lakes that were sampled. In the remaining 27 lakes, brook stickleback were either apparently absent or catches were so small (less than five individuals captured) that a population genetic analysis would have been uninformative. Sampled lakes were generally small, ranging in area from 0.005 to 3.9 km^2^ and from 0.6 to 13 m in maximum depth.

Microsatellite polymorphism varied from five to 21 alleles per locus, with a mean of 12 (Table S2). Populations generally conformed to Hardy–Weinberg expectations (Table S3), although several populations exhibited <20% of loci that significantly differed from Hardy–Weinberg expectations (Table S4). None of the offending loci appeared to have a high propensity to differ from Hardy–Weinberg expectations, with genotype frequencies deviating from Hardy–Weinberg equilibrium in <16% of populations (Table S5). Populations generally did not demonstrate linkage disequilibrium at these loci (data not shown). Several other populations were associated with significant tests for linkage disequilibrium, but these occurred in less than five percent of tests. As in the Hardy–Weinberg tests, the multiple comparison correction method had little influence on the conclusions regarding the tests (see Tables S3 and S4). Therefore, all loci were included for subsequent statistical tests.

Pairwise *F*_ST_ estimates were variable, ranging from a maximum of 0.563 to a minimum of 0.000. The STRUCTURE analysis provided a series of ln(P) values that generally increased as the assumed number of *K* increased, reaching a peak ln(P) at an assumed *K* of 20. The Evanno method also best supported a *K* of 20. Lakes that shared membership in the same cluster were generally located within close proximity of one another (Figure 1).

AMOVA results indicated that molecular variation was influenced by population (Table 2; large scale, *p *<* *.01; medium scale, *p *<* *.01; small scale, *p* < .01), explaining 78.20%, 77.07%, and 79.23% of the total molecular variance in the large‐, medium‐, and small‐watershed tests, respectively. All three tests revealed that among‐lake molecular variation was highly significant, within at least one watershed (*p *<* *.01 at all scales), explaining 16.50%, 13.25%, and 11.32% of the total variance enumerated in the large‐, medium‐, and small‐scale tests, respectively. Significant variation was also explained among watersheds at the large‐, medium‐, and small‐watershed tests (*p *<* *.05, *p *<* *.01, and *p *<* *.01, respectively), although only 5.30%, 9.68%, and 9.45% of the total variation was explained. Pairwise *F*_ST_/1‐*F*_ST_ values did not vary significantly with either pairwise stream network or Euclidean distance (Mantel test, *r *=* *.33, *p *=* *.18; and *r *=* *.29, *p *=* *.11, respectively), indicating the absence of an isolation‐by‐distance pattern.

**Figure 2:**
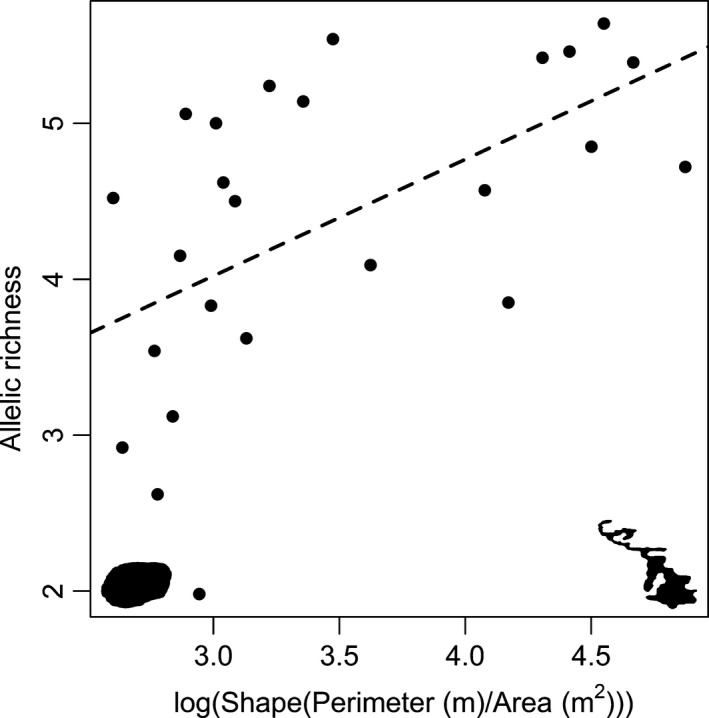
The relationship between lake shape (log(Perimeter (m)^2^/Area (m^2^))) and the allelic richness of brook stickleback populations found within small Central Alberta lakes

**Figure 3:**
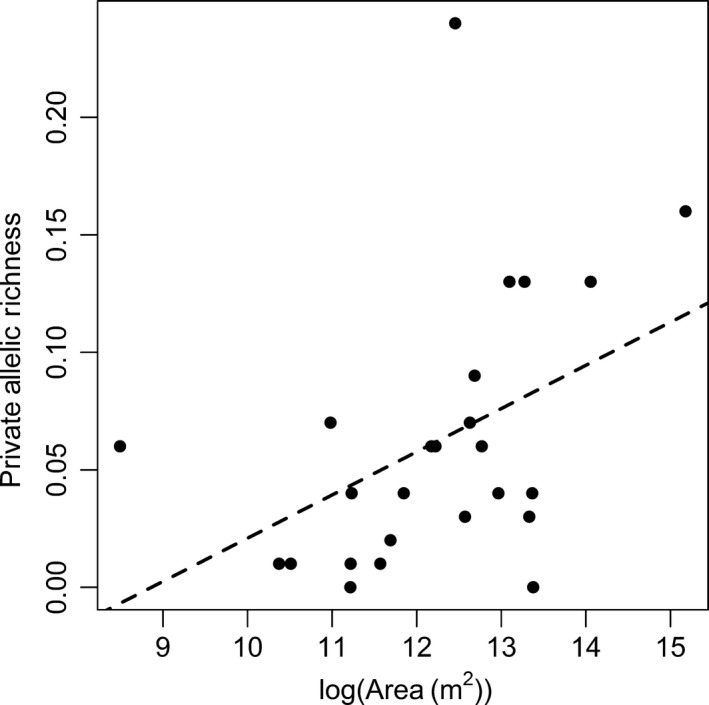
Lake log(Area (m^2^)) and allelic richness of brook stickleback populations

**Figure 4:**
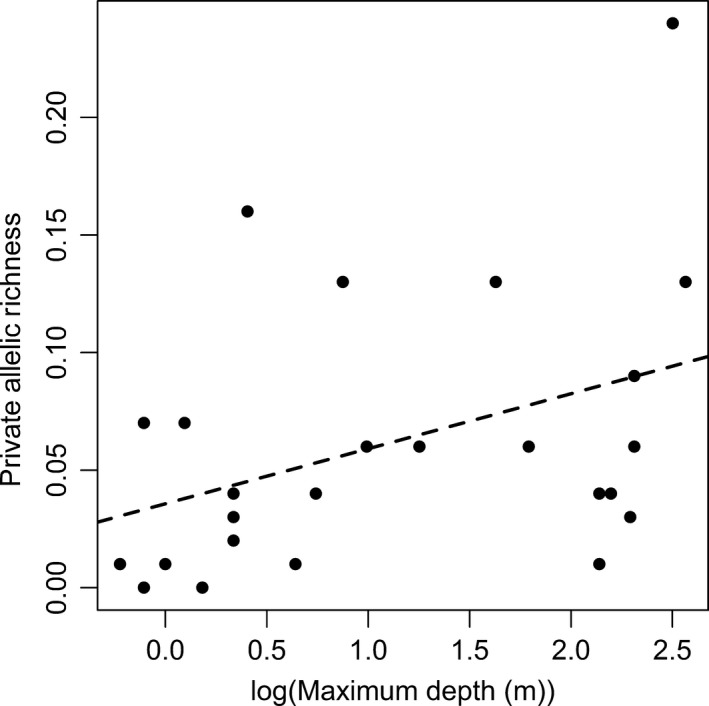
Lake log(Depth (m) and the allelic richness of brook stickleback populations

**Figure 5:**
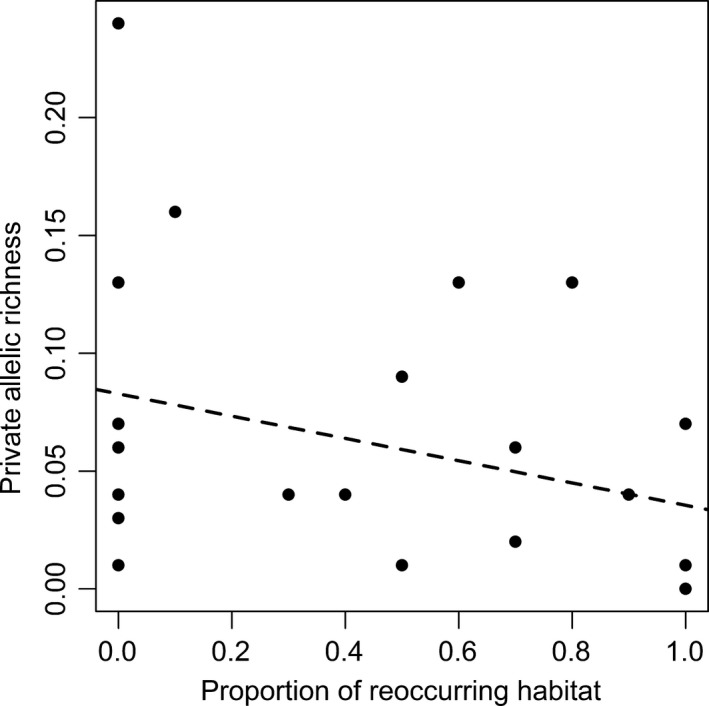
Proportion of reoccurring habitat and allelic richness of brook stickleback populations

Our analyses of the role of spatial features on allelic richness and private allelic richness showed substantial support for “within‐lake” processes being more important than “between‐lake” processes (Table 3), with the best supported models in both analyses categorized as “within‐lake” processes models. The models that were best supported for the analysis of allelic richness appeared to be driven by lake shape (perimeter^2^/lake area) (Figure 2). In contrast, the models that were best supported for the analysis of private allelic richness included area (Figure 3), maximum depth (Figure 4), and the percentage of reoccurring habitat (Figure 5).

**Table 3:** Comparisons of the ability of each model to describe, in separate analyses, private allelic richness (left) and allelic richness (right) of 25 brook stickleback populations in different lakes in Central Alberta using landscape metrics

Analysis of private allelic richness				Analysis of allelic richness			
Model	*K*	AIC_C_	Δ_i_	Model	*K*	AIC_C_	Δ_i_
Area	2	−71.49	0.00	Shape	2	66.43	0.00
Area*Depth	4	−70.53	0.96	Area*Depth + Shape + Radiation + Reoccur	7	68.36	1.93
Depth	2	−70.52	0.96	NearLakes	2	72.55	6.12
Area*Depth + Shape + Radiation + Reoccur	7	−69.65	1.84	Area*Depth + Shape + Slope + NearLakes	7	72.75	6.32
Reoccur	2	−69.21	2.28	Radiation	2	73.04	6.61
Connected	2	−66.76	4.72	Elevation	2	73.13	6.70
Radiation	2	−66.72	4.76	Area	2	73.41	6.98
Slope	2	−66.49	5.00	Slope + NearLakes	3	73.89	7.46
Shape	2	−66.47	5.01	Flow	2	74.12	7.69
NearLakes	2	−66.33	5.15	Connected*Flow	4	74.16	7.73
Flow	2	−66.25	5.23	Area*Depth + Shape + Connected*Flow	8	74.43	8.00
Elevation	2	−66.25	5.24	Slope	2	75.33	8.90
Area*Depth + Shape + Slope + NearLakes	7	−66.09	5.39	Depth	2	75.50	9.07
Area*Depth + Shape + Connected*Flow	8	−65.72	5.76	Reoccur	2	75.57	9.14
Slope + NearLakes	3	−64.53	6.96	Connected	2	75.61	9.19
Slope + Area + NearLakes*Elevation	6	−63.36	8.12	Area*Depth	4	76.31	9.88
Connected*Flow	4	−62.66	8.83	Slope + Area + NearLakes*Elevation	6	76.54	10.11
Connected*Flow + Slope + NearLakes	6	−59.41	12.07	Connected*Flow + Slope + NearLakes	6	76.98	10.56
Connected*Flow*Elevation	8	−56.89	14.60	Connected*Flow*Elevation	8	80.46	14.03
Global Model	17	−55.37	16.12	Global Model	17	87.12	20.69

**Figure 6:**
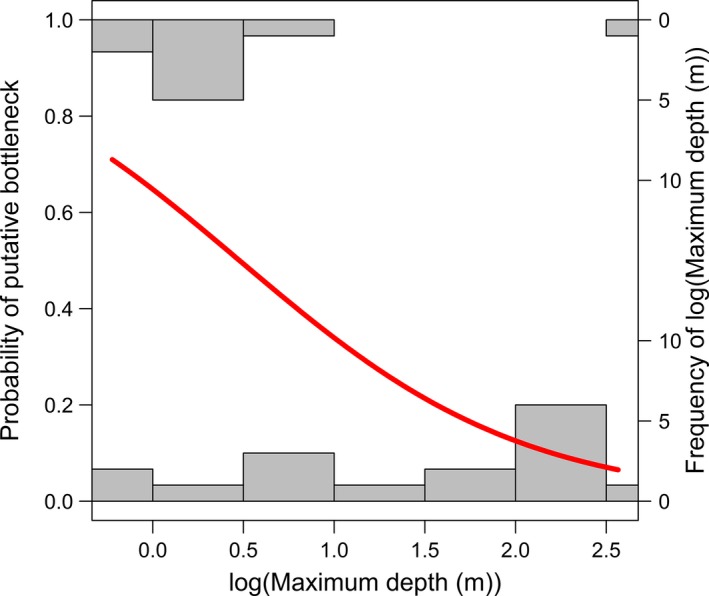
The relationship between log(Maximum Depth (m)) and the Probability of a Putative Bottleneck, with histograms of the frequency of lakes with and without putative bottlenecks Maximum Depth (m)

Outcomes of bottleneck tests were dependent on the assumed mutation mode (data not shown). Tests assuming the IAM detected ten populations that exhibited excess heterozygosity. Tests assuming the TPM detected five populations that exhibited excess heterozygosity, all of which were also significant when the IAM was assumed. Finally, all tests assuming SMM detected heterozygosity deficiency. All other lakes that demonstrated a significant heterozygosity deficiency, and did not have significant excess heterozygosity assuming either the IAM or TPM. Putative bottlenecks were significantly correlated with maximum lake depth (Figure 6; *Z*_23_ = −2.11, *p *=* *.04).

## 4 Discussion

This study characterized the genetic population structure of brook stickleback inhabiting a series of small central Alberta lakes to test predictions associated with the three predominant hypotheses for the role of fragmentation in aquatic environments. We predicted that populations of brook stickleback should exhibit patterns of genetic diversity characteristic of recent population bottlenecks as a result of known winterkills. We measured several landscape metrics and, using an information‐theoretic approach, tested the competing hypotheses that genetic diversity within populations would either be the result of between‐lake processes (i.e., the propensity of a lake and its surroundings to facilitate migration and movement controls genetic diversity) versus within‐lake processes (i.e., features of lakes influence the demographic processes that occur within a lake, and these influence genetic diversity through effective population size and drift). Hierarchal genetic structure patterns between lakes supported the SHM (albeit on a small scale), and demonstrate the continuum between the SHM and DVM, where connectivity is restricted but populations do not appear to be completely isolated. These results are not completely unexpected because, although the focal landscape is highly fragmented, brook stickleback—in contrast to the pupfishes of Death Valley (Turner, 1974)—exhibit relatively minor morphological differentiation between localities (within lineages) and such differences are not thought to be inherited (e.g., Ward & Mclennan, 2009; Zimmerman, 2007). Interestingly, we found most lakes were inhabited by unique populations, with lake diversity influenced predominantly by within‐lake characteristics. Population genetic diversity was also significantly associated with lake basin characteristics. We discuss possible reasons to explain these processes from our data below.

The relationship between populations was not consistent with isolation by distance. No isolation‐by‐distance patterns were detected by stream network or Euclidean distance. STRUCTURE results suggest that most lakes contain groups of individuals that breed more within their group than outside of it. AMOVA results emphasized this conclusion, estimating (on all three scales evaluated) that lake explained approximately 70% of the molecular variation observed. AMOVA results also indicated that a significant but small proportion (<10%) of molecular variance was explained between watersheds at all scales (approximately 15% of variation was explained between lakes within watersheds), indicating that watersheds structure populations together hierarchically. Divergence has evidently been accumulating among watersheds and the watershed genetic divergence is arguably pretty large given the young age of this system that was likely colonized from Mississippian refugium following the retreat of the last glacial maximum (12–15 Kya) (Ward & Mclennan, 2009). Yet, because no isolation‐by‐distance patterns were detected, mistaking hierarchical structure for an isolation‐by‐distance pattern was unlikely (Meirmans, 2012).

The effects of watersheds on molecular variance were relatively small compared to the effects of within‐lake variation. The importance of variability that is explained by population membership is not uncommon and has been documented in other fishes (e.g., Angers, Bernatchez, Angers, & Desgroseillers, 1995; Rogers & Curry, 2004; Shikano, Shimada, Herczeg, & Merilä, 2010; Wilson, Gíslason, & Skúlason, 2004). Such findings may indicate drift or selection acting locally within populations at these spatial scales. It is possible our results may be also biased by the spatial configuration of population groups, with sampling design potentially having significant impacts on the results of other analytical approaches (Schwartz & McKelvey, 2009). Sampled lakes did not always have a replicate (i.e., a second lake) within each watershed group and could not be included in the analysis. The effects of watershed were detected even in immediately neighboring watersheds flowing in different directions, suggesting that our AMOVA was an informative test of hydrological structure.

Both private allelic and allelic richness were best explained by metrics associated with “within‐lake” processes (Table 3). These outcomes are consistent with limited migration and the high degree of structure among populations. In addition, evidence for recent bottlenecks in at least five populations suggests that environmental influences may contribute to within‐lake processes. A candidate for these putative bottlenecks is winter anoxia, which has been shown to cause large population declines in fathead minnows (Danylchuck & Tonn, 2003), which commonly coexist with brook stickleback in the study region. Simulations indicate bottlenecks that occur over only a few generations result in lower detection rates (Williamson‐Natesan, 2005) suggesting that, if winterkills are the mechanism that produces the observed putative bottlenecks, we may be underestimating the prevalence of bottlenecks. The probability of a putative bottleneck, as hypothesized, varied with lake depth (a feature that has been often tied to oxygen decay rates in winter; e.g., Barica & Mathias, 1979; Danylchuck & Tonn, 2003; Mathias & Barica, 1980; Meding & Jackson, 2001, 2003). Because the putative bottleneck association with maximum lake depth is dependent on an assumption of the IAM mutation model, these results warrant further investigation. Although the distribution of lakes that contained a putative bottleneck was biased toward shallow lakes (Figure 6), a relatively even distribution of maximum lakes depths was found in lakes that were not associated with bottlenecks, suggesting that depth may not be the only factor contributing to bottlenecks. Other factors, such macrophyte biomass, have been shown to contribute to anoxia (Meding & Jackson, 2001, 2003). Macrophyte biomass and related variables were absent from our dataset, so we cannot rule out their possible contributions to the observed patterns.

If such population bottlenecks could cause changes in allelic frequencies, an expectation would be that lakes that commonly/recently experience bottlenecks would also have low allelic diversity, but not proportionally low heterozygosity (Nei et al., 1975). Several “within‐lake” processes metrics were demonstrated to vary with allelic diversity, and may be representative of the propensity of a lake to experience a bottleneck. Such a disturbance regime could also contribute the high degrees of differentiation observed, as large stochastic changes in population size could accelerate processes such as genetic drift (Chakraborty & Nei, 1977; Hedrick, 1999) and potentially local adaptation (Koskinen, Haugen, & Primmer, 2002). Indeed, we discovered several populations deviating from HWE (see Supplemental Information). Factors associated with deviations from HWE only in certain samples include the Wahlund effect, small Ne, genotyping errors, or sex‐specific differences (Waples, 2015). Our analyses suggest that small effective population sizes due to winterkill may be the most likely source contributing to these patterns of genetic variation, but future studies will want to explore this possibility further with genomewide data (Waples, 2015). Given that it remains difficult to empirically identify null alleles, estimating null alleles may also obscure results when using HWE departures to assess the roles of mutation, selection, and drift in generating and maintaining patterns of diversity. Sequencing microsatellites with next‐generation sequencing will likely improve estimates.

In addition, lake shapes of 20 of the 25 lakes were manually measured, while 5 were collected from government sources (Yellowhead, Beaver, Birch, Fiesta, and Strubel). Thus, more precise measures of lake could overcome this caveat to improve estimation of lake shape variables. Nonetheless, these patterns collectively demonstrate the importance and impact of watershed scale in structuring brook stickleback genetic variation.

## 5 General Conclusions and Future Directions

Brook stickleback populations appear to be influenced by within‐lake characteristics, although weak hierarchical structuring by small‐ and medium‐scale watersheds was apparent, suggesting some gene flow between nearby populations. Gene flow between brook stickleback populations is likely the result of dispersal through the stream network to nearby lakes. This study supports the findings of other studies demonstrating the importance of hydrological structure in determining population structure (e.g., Castric et al., 2001; Huey et al., 2008; Meeuwig et al., 2010).

Landscape fragmentation has been linked to decreased (e.g., Keller & Largiadèr, 2003; Schtickzelle, Mennechez, & Baguette, 2006; Stow, Sunnucks, Briscoe, & Gardner, 2001) and increased migration estimates (e.g., Peacock & Smith, 1997) between populations/patches. The landscape genetic patterns documented here strongly suggest that in highly fragmented landscapes, where gene flow is restricted, within‐lake features are especially important in determining a population's genetic diversity. Overall, our results suggest that landscape fragmentation (and resulting low gene flow) can accelerate genetic drift, facilitate divergence between populations, reduce the genetic diversity found within populations, and potentially contribute to reductions in estimates of gene flow. The emphasis on gene flow in the landscape genetics literature is important, and critical to understanding evolutionary processes in contemporary landscapes (Storfer et al., 2010). However, the mechanisms that influence genetic drift in a landscape context need further attention for a full understanding of how landscapes, and the processes that occur within them, influence evolution.

## Conflict of Interest

None declared.
