# Supplementary material for: Watershed characteristics shape the landscape genetics of brook stickleback (Culaea inconstans) in shallow prairie lakes
Source: Ecol Evol. 2017 Mar 28;7(9):3067–79. doi: 10.1002/ece3.2885 (PMC5415534; doi:10.1002/ece3.2885)
Supplement: Supplementary file 1 [file ECE3-7-3067-s001.docx]

Table S1: Brook stickleback capture statistics for the 52 lakes sampled.

| Lake Name | Abbreviation | Year Sampled | Latitude (decimal degrees) | Longitude (decimal degrees) | Number of Traps Set | Effort (Trap Hours) |
| --- | --- | --- | --- | --- | --- | --- |
| Adams | ADA | 2011 | 52.07 | 114.48 | 20 | 468.0 |
| Arden | ARD | 2011 | 52.07 | 114.58 | 20 | 372.0 |
| Beaver | BEA | 2011 | 52.01 | 114.79 | 20 | 425.3 |
| Big Arden | BAR | 2011 | 52.07 | 114.58 | 20 | 453.0 |
| Big North | BN | 2012 | 52.26 | 114.75 | 20 | 432.0 |
| Birch | BIR | 2011 | 52.01 | 114.86 | 21 | 446.6 |
| Bowden | BOW | 2012 | 51.94 | 114.07 | 20 | 329.3 |
| Cowboy | COW | 2011 | 52.01 | 114.67 | 20 | 436.0 |
| Crammond | CRA | 2011 | 51.95 | 114.64 | 20 | 370.0 |
| Dickson Dam Trout Pond (DDTP) | DDT | 2011 | 52.01 | 114.31 | 20 | 376.7 |
| Dodds | DOD | 2012 | 52.03 | 113.96 | 20 | 364.7 |
| Dogleg | DL | 2011 | 51.99 | 114.73 | 20 | 349.7 |
| Eckford | ECK | 2012 | 51.99 | 114.69 | 20 | 383.3 |
| Fiesta | FIE | 2011 | 52.00 | 114.73 | 16 | 344.0 |
| Frog Pond | FP | 2012 | 52.25 | 114.56 | 20 | 456.7 |
| Gasplant | GAS | 2011 | 51.99 | 114.76 | 18 | 357.3 |
| Gunrange | GUN | 2011 | 51.99 | 114.72 | 19 | 437.0 |
| Honeysuckle | HON | 2012 | 52.25 | 114.76 | 19 | 437.0 |
| Horseshoe | HS | 2011 | 52.00 | 114.82 | 20 | 391.0 |
| Johnson | JON | 2011 | 51.96 | 114.71 | 19 | 431.6 |
| Johnson East | JE | 2011 | 51.97 | 114.70 | 20 | 394.0 |
| Johnson West | JW | 2011 | 51.97 | 114.71 | 20 | 436.7 |
| Little Yellowhead | LYH | 2011 | 51.96 | 114.81 | 20 | 398.7 |
| Mitchell | MIT | 2012 | 52.22 | 115.01 | 20 | 324.0 |
| Napoleon | NAP | 2012 | 52.03 | 113.97 | 20 | 397.0 |
| North Split | NS | 2012 | 52.27 | 114.57 | 20 | 426.0 |
| Oz | OZ | 2012 | 52.00 | 114.54 | 20 | 421.0 |
| Pike Lake | PIL | 2011 | 52.08 | 114.16 | 20 | 391.7 |
| South Frog Pond | SFP | 2011 | 52.25 | 114.55 | 20 | 324.7 |
| Split | SPL | 2011 | 52.15 | 114.55 | 20 | 432.0 |
| Stauffer | STA | 2011 | 52.20 | 114.63 | 20 | 346.7 |
| Struble | STR | 2012 | 52.20 | 115.00 | 20 | 286.3 |
| Sunglache | SUN | 2011 | 52.13 | 114.41 | 20 | 469.7 |
| Teal | TEA | 2011 | 51.98 | 114.69 | 20 | 377.0 |
| Unnamed 04 | U04 | 2011 | 52.16 | 114.58 | 20 | 467.7 |
| Unnamed 16 | U16 | 2012 | 52.09 | 114.10 | 19 | 366.1 |
| Unnamed 17 | U17 | 2011 | 52.06 | 114.46 | 20 | 397.7 |
| Unnamed 19 | U19 | 2011 | 52.07 | 114.45 | 20 | 406.3 |
| Unnamed 20 | U20 | 2011 | 52.06 | 114.79 | 20 | 401.7 |
| Unnamed 21 | U21 | 2011 | 52.01 | 114.61 | 20 | 426.0 |
| Unnamed 22 | U22 | 2011 | 52.04 | 114.66 | 20 | 468.7 |
| Unnamed 24 | U24 | 2011 | 52.04 | 114.81 | 20 | 388.3 |
| Unnamed 25 | U25 | 2011 | 52.01 | 114.83 | 20 | 421.3 |
| Unnamed 30 | U30 | 2011 | 52.00 | 114.72 | 20 | 436.7 |
| Unnamed 36 | U36 | 2011 | 51.99 | 114.85 | 20 | 368.0 |
| Unnamed 37 | U37 | 2011 | 51.99 | 114.82 | 20 | 406.7 |
| Unnamed 38 | U38 | 2011 | 51.99 | 114.69 | 20 | 420.7 |
| Unnamed 42 | U42 | 2011 | 51.98 | 114.80 | 20 | 413.7 |
| Unnamed 43 | U43 | 2011 | 51.97 | 114.73 | 20 | 485.0 |
| Unnamed 50 | U50 | 2011 | 51.95 | 114.71 | 20 | 312.7 |
| Unnamed 51 | U51 | 2011 | 52.20 | 114.53 | 20 | 414.7 |
| Yellowhead | YEL | 2011 | 51.96 | 114.80 | 20 | 403.0 |

Table S2: Microsatellite primers tested for amplification and polymorphism, species that they were originally characterized for, forward and reverse sequences, amplification success in brook stickleback, number of alleles identified (N_A_, if selected for genotyping), observed heterozygosity (H_o_), and the expected heterozygosity of genotyped markers (H_e_). An asterisk associated with allele size range indicates that the size range includes a M13 tag.

| **Primer** | **Species of Origin** | **Primer Sequence** | **Successful Amplification** | **N_A_** | **Allele Range (BP)** | **H_o_** | **H_e_** |
| --- | --- | --- | --- | --- | --- | --- | --- |
| GAC2111_a_ | *Gasterosteus aculeatus* | GTAGAGCACTTGAACTTGAACTG | Yes | - |  |  |  |
|  |  | GACGTAGATTGTGGATGTAGAGG |  |  |  |  |  |
| GAC4174_a_ | *Gasterosteus aculeatus* | CCGCGATGATGAGAGTG | Yes | - |  |  |  |
|  |  | GTGAAATGCGACAGATGATG |  |  |  |  |  |
| STN5_b_ | *Gasterosteus aculeatus* | CACACATACACTCACACACGC | No | - |  |  |  |
|  |  | CCAGTAGCATTCAACCAAAGC |  |  |  |  |  |
| STN14_b_ | *Gasterosteus aculeatus* | GTGAACCAAACTCATAACAGCG | Yes | - |  |  |  |
|  |  | CTTGTCCCTGGATGAGAACC |  |  |  |  |  |
| STN33_b_ | *Gasterosteus aculeatus* | GCTTACAGCCATTACGTGGG | No | - |  |  |  |
|  |  | GAGACAATTAAGCAACGTGGG |  |  |  |  |  |
| STN47_b_ | *Gasterosteus aculeatus* | AAGGAAGGGAAGCAGTGAGC | Yes | - |  |  |  |
|  |  | ACGGGTTAGAAGTTCCTCCC |  |  |  |  |  |
| STN58_b_ | *Gasterosteus aculeatus* | GTAAGCTAAGGCAAACCGGG | Yes | - |  |  |  |
|  |  | GTTCATCTGATCCGTGTCCC |  |  |  |  |  |
| STN65_b_ | *Gasterosteus aculeatus* | ATTGAACGACTGCTTTGTGC | Yes | - |  |  |  |
|  |  | TTGTACCGCCAGATGAAAGC |  |  |  |  |  |
| STN83_b_ | *Gasterosteus aculeatus* | CTCTGCTGGCAGAAATGGG | Yes | - |  |  |  |
|  |  | CGTCAGATACATTCAAGAACGC |  |  |  |  |  |
| STN96_b_ | *Gasterosteus aculeatus* | ACACCTTCGGCTCCATATCC | Yes | - |  |  |  |
|  |  | CGCAGCTCTCTGCTTTGC |  |  |  |  |  |
| STN101_b_ | *Gasterosteus aculeatus* | CAGCTTTGTGTTCTGGGAGG | No | - |  |  |  |
|  |  | GGAGGCTCTAATGAATCAGCC |  |  |  |  |  |
| STN106_b_ | *Gasterosteus aculeatus* | TCACCGTGAGTTACACACCC | Yes | - |  |  |  |
|  |  | GTTCAATTCCTCCATCACGG |  |  |  |  |  |
| STN110_b_ | *Gasterosteus aculeatus* | AGACAAACTCATGTAACAGCCC | Yes | - |  |  |  |
|  |  | ACCTGGGTGCTTCAATGC |  |  |  |  |  |
| STN114_b_ | *Gasterosteus aculeatus* | GAGTTCCTAACCGTCCTCCC | Yes | - |  |  |  |
|  |  | TGTCCATAAACAAACCCACG |  |  |  |  |  |
| STN137_b_ | *Gasterosteus aculeatus* | TAGGTTGTTCTGTGACCCGC | Yes | - |  |  |  |
|  |  | GATGGACGGGAAGATAAACG |  |  |  |  |  |
| STN150_b_ | *Gasterosteus aculeatus* | TCCTGCACACAGTCAACTCC | Yes | - |  |  |  |
|  |  | GTTGCTATGGAGACAGTGGC |  |  |  |  |  |
| STN159_b_ | *Gasterosteus aculeatus* | TCAGGATGGTCTCCTCTTCG | Yes | - |  |  |  |
|  |  | GAAAGCCTCCGAGAGAATCC |  |  |  |  |  |
| STN166_b_ | *Gasterosteus aculeatus* | ATGCTCTCCCTGTGATCTCC | Yes | - |  |  |  |
|  |  | TCACTCTGACTGATTGCTCCC |  |  |  |  |  |
| STN168_b_ | *Gasterosteus aculeatus* | AAAGTGCATCTTTGGGTGC | Yes | 8 | 187-201 | 0.397 | 0.539 |
|  |  | TGATCAATACAGCTTGTCAGCC |  |  |  |  |  |
| STN173_b_ | *Gasterosteus aculeatus* | ACCACTTTGATTGGAATGGG | No | - |  |  |  |
|  |  | GACGAAATGTACAGCACATCC |  |  |  |  |  |
| STN175_b_ | *Gasterosteus aculeatus* | TCTACATCATCTGTTACCACGG | No | - |  |  |  |
|  |  | TTGCATGAGCGTGTAAAACC |  |  |  |  |  |
| STN177_b_ | *Gasterosteus aculeatus* | GTGTTGGCAGTTATCAGGGC | Yes | - |  |  |  |
|  |  | ACCTATGCTGCTCCAAGACC |  |  |  |  |  |
| STN179_b_ | *Gasterosteus aculeatus* | CATCGGAACATCTGCTATGC | Yes | - |  |  |  |
|  |  | CTCCCTCTCATTTCACACGG |  |  |  |  |  |
| STN183_b_ | *Gasterosteus aculeatus* | CAGCCAGGGTAGTAGAAGCG | Yes | - |  |  |  |
|  |  | AGCTAACCGGTGTTCTCAGC |  |  |  |  |  |
| STN186_b_ | *Gasterosteus aculeatus* | CTCACTTCCCAAATGTCACG | Yes | 13 | 182-228 | 0.618 | 0.718 |
|  |  | TACCTGGCAGCCTAATGACC |  |  |  |  |  |
| STN188_b_ | *Gasterosteus aculeatus* | AGCCAAACGGAGGAAGAGC | Yes | - |  |  |  |
|  |  | GTCTCTCTGCTGTCGCTGC |  |  |  |  |  |
| STN196_b_ | *Gasterosteus aculeatus* | GAGATGGCAATGAAGATGCC | Yes | - |  |  |  |
|  |  | ACACATACACAGTCGTGGGC |  |  |  |  |  |
| STN198_b_ | *Gasterosteus aculeatus* | AGAAGGCCATCCCTTTGG | Yes | - |  |  |  |
|  |  | AAAGAACGAACGAGTGAGCG |  |  |  |  |  |
| STN200_b_ | *Gasterosteus aculeatus* | GTCTCTTCAGGCCAAAGTCG | Yes | - |  |  |  |
|  |  | TATTGATTTATGGCTCCGGG |  |  |  |  |  |
| STN210_c_ | *Gasterosteus aculeatus* | TGAGGAGAGATGTTGAGCCG | Yes | - |  |  |  |
|  |  | TCGTGTTTCCTACCAAGATGG |  |  |  |  |  |
| STN211_c_ | *Gasterosteus aculeatus* | ACAACTCTTCCTTTGGCTGG | Yes | - |  |  |  |
|  |  | ATAAATCCAGGCCACACACG |  |  |  |  |  |
| STN216_c_ | *Gasterosteus aculeatus* | TGTGCAGTAGAGCAACAGCC | Yes | - |  |  |  |
|  |  | TGTTTCTGGCAGTAGGGTCC |  |  |  |  |  |
| STN217_c_ | *Gasterosteus aculeatus* | GATGGACTGTGGTAGAGCCC | Yes | 18 | 151-197 | 0.750 | 0.852 |
|  |  | AACATGAAGGATCACACCTGC |  |  |  |  |  |
| STN222_c_ | *Gasterosteus aculeatus* | TTCCATTTAGATGAAGGCGG | Yes | - |  |  |  |
|  |  | AAGCAGTGGAGAGTTGACCC |  |  |  |  |  |
| STN226_d_ | *Gasterosteus aculeatus* | AAGATACGGACGCACTGAGC | No | - |  |  |  |
|  |  | TGAGTGAAGGGCTGAGATGG |  |  |  |  |  |
| STN241_d_ | *Gasterosteus aculeatus* | GACCTCCAGAACCAGGAAGG | No | - |  |  |  |
|  |  | CTTTACCAAGGTGAGGGACG |  |  |  |  |  |
| STN242_d_ | *Gasterosteus aculeatus* | GCACATTATGTTGCTGCTTCC | Yes | - |  |  |  |
|  |  | CAGCTGTGCTGAACATTTGC |  |  |  |  |  |
| STN244_d_ | *Gasterosteus aculeatus* | GGTCATCCAAAGTCTGTCGC | Yes | - |  |  |  |
|  |  | TTCACTATATTGGCCTCGCC |  |  |  |  |  |
| STN250_d_ | *Gasterosteus aculeatus* | TGGAAAGCACTTGGATGAGC | No | - |  |  |  |
|  |  | TCAGTTCTTTGAGGCCTTCC |  |  |  |  |  |
| STN265_d_ | *Gasterosteus aculeatus* | CGTAGTGTGAAACCACAGGC | Yes | 18 | 457-499 | 0.667 | 0.841 |
|  |  | TGTGTCCACAGAGATGAGGC |  |  |  |  |  |
| STN277_d_ | *Gasterosteus aculeatus* | TGAGTTGTGCAGGAATCTGG | Yes | - |  |  |  |
|  |  | ACGTGATGTGGTAGTGGTGG |  |  |  |  |  |
| STN289_d_ | *Gasterosteus aculeatus* | CACTGTTAAAGCACTTGTGTGG | No | - |  |  |  |
|  |  | TGGTCTTCTTACAGGCTCGC |  |  |  |  |  |
| STN301_d_ | *Gasterosteus aculeatus* | GTTGGTATATTTGCTGCGGG | Yes | 21 | 173-219 | 0.761 | 0.897 |
|  |  | GTGTGCATATTGCCCTTGC |  |  |  |  |  |
| STN304_d_ | *Gasterosteus aculeatus* | TCTAGCTCTTCTTCCAGGGC | No | - |  |  |  |
|  |  | AGATGGCCCAGTTATGAACG |  |  |  |  |  |
| STN319_d_ | *Gasterosteus aculeatus* | CCCTCACTGATAACTAGGCCC | Yes | - |  |  |  |
|  |  | AACGAGCGACACGATAGAGG |  |  |  |  |  |
| STN324_d_ | *Gasterosteus aculeatus* | GACAACTCACCCTAGTGCGG | No | - |  |  |  |
|  |  | CATGTAAGCAGCAAAGCACG |  |  |  |  |  |
| STN328_d_ | *Gasterosteus aculeatus* | GTCAGCGCTTGAGAAGAACC | Yes | 9 | 142-162 | 0.067 | 0.229 |
|  |  | TGAGAGGAGAGGCTGAGAGG |  |  |  |  |  |
| STN337_e_ | *Gasterosteus aculeatus* | ACACAAACCTTTAGCACTGG | No | - |  |  |  |
|  |  | AGTGTGACGTCCAAAATAGG |  |  |  |  |  |
| STN344_d_ | *Gasterosteus aculeatus* | TTTGTTGGGATCTGGAGACG | No | - |  |  |  |
|  |  | GAGCTCTTCAAGCTGGTTCC |  |  |  |  |  |
| PUN19_f_ | *Pungitius pungitius* | TGCAATCTCTTCTGTCGTGC | Yes | 5 | 412-420* | 0.152 | 0.539 |
|  |  | AGCCTCTGACAAACTGAGCC |  |  |  |  |  |
| PUN20_f_ | *Pungitius pungitius* | GACTCCAGAAACACACTAAGGC |  | - |  |  |  |
|  |  | TCACAGGGCTTAAGAATGCC |  |  |  |  |  |
| PUN44_f_ | *Pungitius pungitius* | GTGAGTAACTCGCTGGTGGC | Yes | - |  |  |  |
|  |  | TATTCTTCCACGCTTGACCC |  |  |  |  |  |
| PUN45_f_ | *Pungitius pungitius* | AGGGTTCATCTGTGAATGCC |  | - |  |  |  |
|  |  | CATTTGTCCCAACATCTCCC |  |  |  |  |  |
| PUN60_f_ | *Pungitius pungitius* | AGACGTGATACTGTGAGCGG |  | - |  |  |  |
|  |  | TCCACCACGTTACAGGGG |  |  |  |  |  |
| PUN61_f_ | *Pungitius pungitius* | CTTATCTGAGCTGTCAGCGG | Yes | 15 | 155-183* | 0.658 | 0.834 |
|  |  | GGATCTGTGGAAAAGGTAGCC |  |  |  |  |  |
| PUN78_f_ | *Pungitius pungitius* | AGACAGGTAGAGGCATCCCC |  | - |  |  |  |
|  |  | GACTGAACTCAGCAGGAGGG |  |  |  |  |  |
| PUN98_f_ | *Pungitius pungitius* | GGCAGTCACGTAGTTTTCTGG | Yes | 10 | 163-183* | 0.428 | 0.734 |
|  |  | GGAATCTGAGTGAAGAGGCG |  |  |  |  |  |
| PUN114_f_ | *Pungitius pungitius* | ATGTGAGGGATGACGAGAGC |  | - |  |  |  |
|  |  | TTGCGCAATAATGACTCAGG |  |  |  |  |  |
| PUN117_f_ | *Pungitius pungitius* | ACGCTGTGTCTGCAAGAATG |  | - |  |  |  |
|  |  | CCGATAGAGGGAAAATACACAC |  |  |  |  |  |
| PUN134_f_ | *Pungitius pungitius* | GTAAAGTCCAGCCCAGACGG |  | - |  |  |  |
|  |  | GTACTCATGAACACAGGGCG |  |  |  |  |  |
| PUN157_f_ | *Pungitius pungitius* | ACATGCCCTTTGTCAGTGC |  | - |  |  |  |
|  |  | CGTTTCACAGTCACTTCACG |  |  |  |  |  |
| PUN196_f_ | *Pungitius pungitius* | TGTGGAATGCCTCGAATACC | Yes | 6 | 209-221* | 0.459 | 0.661 |
|  |  | TGCTGTTTCTCTTCTCTGTCTC |  |  |  |  |  |
| PUN203_f_ | *Pungitius pungitius* | GTAAAACGCGTCTGAGTGCC |  | - |  |  |  |
|  |  | TTTCCTCAAAGTTATGGCCG |  |  |  |  |  |
| PUN210_f_ | *Pungitius pungitius* | AGGAAAATTCAGGGGAAAGG |  | - |  |  |  |
|  |  | CCAACGTGCAGTGATGACG |  |  |  |  |  |
| PUN212_f_ | *Pungitius pungitius* | TGCACAAAACAAACAGACCC |  | - |  |  |  |
|  |  | GTGTGATGCTCACAGAACGC |  |  |  |  |  |
| PUN217_f_ | *Pungitius pungitius* | CAGGTCAATCAGTGGAACCG | Yes | 10 | 219-237* | 0.562 | 0.770 |
|  |  | CCACACAGAGCTTGCTCCC |  |  |  |  |  |
| PUN255_f_ | *Pungitius pungitius* | TGCTGTCTGTTTCTGTTGGG |  | - |  |  |  |
|  |  | AACAGTTAGGGAAGGGAGGC |  |  |  |  |  |
| PUN261_f_ | *Pungitius pungitius* | GCCACTACACCACCTACACC |  | - |  |  |  |
|  |  | CAAGCCTGGAGAGTAATTCG |  |  |  |  |  |

a: Largiadèr et al. (1999)

b: Peichel et al. (2001)

c: Colosimo et al. (2004)

d: Peichel, Oghi, Cole and Kingsley (unpublished)

e: Knecht et al. (2007)

f: Shapiro et al. (2009)

Table S3. Observed and expected heterozygosity for each locus in each population sampled

| **Adams** | | |
| --- | --- | --- |
| Locus | Observed Heterozygosity | Expected Heterozygosity |
| PUN19 | 0.34146 | 0.352 |
| PUN196 | 0.71429 | 0.69441 |
| PUN217 | 0.65854 | 0.61999 |
| PUN61 | 0.725 | 0.73608 |
| PUN98 | 0.38235 | 0.68174 |
| STN168 | 0.48571 | 0.48075 |
| STN186 | 0.54286 | 0.50807 |
| STN217 | 0.6 | 0.69304 |
| STN265 | 0.73684 | 0.7214 |
| STN301 | 0.89744 | 0.84282 |
| STN328 | 0.12195 | 0.11593 |

| **Arden** | | |
| --- | --- | --- |
| Locus | Observed Heterozygosity | Expected Heterozygosity |
| PUN19 | 0.5 | 0.52437 |
| PUN196 | 0.20455 | 0.22126 |
| PUN217 | 0.59091 | 0.48824 |
| PUN61 | 0.54545 | 0.61677 |
| PUN98 | 0.16279 | 0.39808 |
| STN168 | 0.38636 | 0.36755 |
| STN186 | 0.27273 | 0.30486 |
| STN217 | 0.81818 | 0.62356 |
| STN265 | 0.48718 | 0.48052 |
| STN301 | 0.12195 | 0.11864 |
| STN328 | 0.61364 | 0.60005 |

| **Beaver** | | |
| --- | --- | --- |
| Locus | Observed Heterozygosity | Expected Heterozygosity |
| PUN19 | 0.51852 | 0.42488 |
| PUN196 | 0.66667 | 0.57296 |
| PUN217 | 0.625 | 0.61558 |
| PUN61 | 0.78125 | 0.75248 |
| PUN98 | 0.16667 | 0.21638 |
| STN168 | 0.67742 | 0.5505 |
| STN186 | 0.68966 | 0.60738 |
| STN217 | 0.64516 | 0.78213 |
| STN265 | 0.75 | 0.82589 |
| STN301 | 0.75758 | 0.74825 |
| STN328 | 0.21212 | 0.1972 |

| **Big North** | | |
| --- | --- | --- |
| Locus | Observed Heterozygosity | Expected Heterozygosity |
| PUN19 | Monomorphic | |
| PUN196 | 0.6 | 0.66742 |
| PUN217 | 0.71111 | 0.69538 |
| PUN61 | 0.91111 | 0.8362 |
| PUN98 | 0.46512 | 0.59863 |
| STN168 | 0.51111 | 0.49638 |
| STN186 | 0.88889 | 0.79276 |
| STN217 | 0.93333 | 0.83945 |
| STN265 | 0.81081 | 0.75935 |
| STN301 | 0.86364 | 0.90517 |
| STN328 | Monomorphic | |

| **Birch** | | |
| --- | --- | --- |
| Locus | Observed Heterozygosity | Expected Heterozygosity |
| PUN19 | 0 | 0.05062 |
| PUN196 | 0.65217 | 0.63163 |
| PUN217 | 0.56522 | 0.53153 |
| PUN61 | 0.43478 | 0.46058 |
| PUN98 | 0.34783 | 0.48184 |
| STN168 | 0.41304 | 0.50334 |
| STN186 | 0.59091 | 0.53736 |
| STN217 | 0.64444 | 0.6779 |
| STN265 | 0.68293 | 0.68714 |
| STN301 | 0.8913 | 0.86598 |
| STN328 | Monomorphic | |

| **Cowboy** | | |
| --- | --- | --- |
| Locus | Observed Heterozygosity | Expected Heterozygosity |
| PUN19 | 0.16216 | 0.42466 |
| PUN196 | 0.23684 | 0.21158 |
| PUN217 | 0.55263 | 0.60035 |
| PUN61 | 0.54054 | 0.58941 |
| PUN98 | 0.64865 | 0.75787 |
| STN168 | 0.21053 | 0.19614 |
| STN186 | 0.51351 | 0.46242 |
| STN217 | 0.67568 | 0.80304 |
| STN265 | 0.80556 | 0.78365 |
| STN301 | 0.89189 | 0.89559 |
| STN328 | Monomorphic | |

| **Dickson Dam Trout Pond** | | |
| --- | --- | --- |
| Locus | Observed Heterozygosity | Expected Heterozygosity |
| PUN19 | Monomorphic | |
| PUN196 | 0.73913 | 0.68849 |
| PUN217 | 0.04348 | 0.04324 |
| PUN61 | 0.21739 | 0.4269 |
| PUN98 | 0.46667 | 0.42472 |
| STN168 | 0.34783 | 0.344 |
| STN186 | 0.71739 | 0.77664 |
| STN217 | 0.77778 | 0.69713 |
| STN265 | 0.575 | 0.69842 |
| STN301 | 0.6 | 0.65194 |
| STN328 | Monomorphic | |

| **Dodds** | | |
| --- | --- | --- |
| Locus | Observed Heterozygosity | Expected Heterozygosity |
| PUN19 | Monomorphic | |
| PUN196 | 0.6 | 0.51808 |
| PUN217 | 0.22581 | 0.20888 |
| PUN61 | 0.3871 | 0.40137 |
| PUN98 | 0.32258 | 0.31729 |
| STN168 | 0.6129 | 0.50608 |
| STN186 | 0.51613 | 0.50767 |
| STN217 | 0.74194 | 0.66896 |
| STN265 | 0.48387 | 0.633 |
| STN301 | 0.41935 | 0.52459 |
| STN328 | Monomorphic | |

| **Eckford** | | |
| --- | --- | --- |
| Locus | Observed Heterozygosity | Expected Heterozygosity |
| PUN19 | 0.04167 | 0.04123 |
| PUN196 | 0.16667 | 0.15789 |
| PUN217 | 0.75 | 0.72193 |
| PUN61 | 0.64444 | 0.67516 |
| PUN98 | 0.52083 | 0.56557 |
| STN168 | 0.20833 | 0.19232 |
| STN186 | 0.60417 | 0.66754 |
| STN217 | 0.8125 | 0.8068 |
| STN265 | 0.62791 | 0.69822 |
| STN301 | 0.93478 | 0.82656 |
| STN328 | 0 | 0.04395 |

| **Fiesta** | | |
| --- | --- | --- |
| Locus | Observed Heterozygosity | Expected Heterozygosity |
| PUN19 | 0.275 | 0.24968 |
| PUN196 | 0.2093 | 0.1896 |
| PUN217 | 0.55814 | 0.542 |
| PUN61 | 0.62791 | 0.64405 |
| PUN98 | 0.69767 | 0.7015 |
| STN168 | 0.16279 | 0.19644 |
| STN186 | 0.62791 | 0.57428 |
| STN217 | 0.88372 | 0.83283 |
| STN265 | 0.65714 | 0.77723 |
| STN301 | 0.87179 | 0.90376 |
| STN328 | 0.025 | 0.025 |

| **Frog Pond** | | |
| --- | --- | --- |
| Locus | Observed Heterozygosity | Expected Heterozygosity |
| PUN19 | 0.1875 | 0.51389 |
| PUN196 | 0.6875 | 0.65427 |
| PUN217 | 0.59375 | 0.76488 |
| PUN61 | 0.5625 | 0.68651 |
| PUN98 | 0.65625 | 0.56101 |
| STN168 | 0.40625 | 0.52629 |
| STN186 | 0.59375 | 0.61657 |
| STN217 | 0.84375 | 0.82044 |
| STN265 | 0.625 | 0.75744 |
| STN301 | 0.60714 | 0.88247 |
| STN328 | Monomorphic | |

| **Gasplant** | | |
| --- | --- | --- |
| Locus | Observed Heterozygosity | Expected Heterozygosity |
| PUN19 | 0.48718 | 0.48518 |
| PUN196 | 0.625 | 0.56582 |
| PUN217 | 0.675 | 0.63766 |
| PUN61 | 0.75 | 0.6769 |
| PUN98 | 0.19512 | 0.20476 |
| STN168 | 0.62162 | 0.58682 |
| STN186 | 0.61111 | 0.5716 |
| STN217 | 0.60976 | 0.76031 |
| STN265 | 0.63636 | 0.74126 |
| STN301 | 0.73171 | 0.76754 |
| STN328 | 0.2 | 0.18829 |

| **Gunrange** | | |
| --- | --- | --- |
| Locus | Observed Heterozygosity | Expected Heterozygosity |
| PUN19 | 0.25 | 0.22887 |
| PUN196 | 0.38889 | 0.31768 |
| PUN217 | 0.63636 | 0.56503 |
| PUN61 | 0.70588 | 0.65628 |
| PUN98 | 0.57143 | 0.6294 |
| STN168 | 0.24242 | 0.22238 |
| STN186 | 0.54545 | 0.54452 |
| STN217 | 0.71429 | 0.84928 |
| STN265 | 0.56667 | 0.81582 |
| STN301 | 0.87879 | 0.9049 |
| STN328 | Monomorphic | |

| **Honeysuckle** | | |
| --- | --- | --- |
| Locus | Observed Heterozygosity | Expected Heterozygosity |
| PUN19 | 0.06977 | 0.06813 |
| PUN196 | 0.65116 | 0.65363 |
| PUN217 | 0.74419 | 0.68509 |
| PUN61 | 1 | 0.84679 |
| PUN98 | 0.4878 | 0.53809 |
| STN168 | 0.53488 | 0.56224 |
| STN186 | 0.74419 | 0.7844 |
| STN217 | 1 | 0.84679 |
| STN265 | 0.80488 | 0.76995 |
| STN301 | 0.92683 | 0.90545 |
| STN328 | Monomorphic | |

| **Johnson West** | | |
| --- | --- | --- |
| Locus | Observed Heterozygosity | Expected Heterozygosity |
| PUN19 | 0.02222 | 0.02222 |
| PUN196 | 0.34783 | 0.47301 |
| PUN217 | 0.76087 | 0.73889 |
| PUN61 | 0.71739 | 0.68849 |
| PUN98 | 0.6087 | 0.76159 |
| STN168 | 0.41304 | 0.40158 |
| STN186 | 0.63636 | 0.60711 |
| STN217 | 0.86667 | 0.8769 |
| STN265 | 0.53488 | 0.66211 |
| STN301 | 0.87179 | 0.85981 |
| STN328 | Monomorphic | |

| **Oz** | | |
| --- | --- | --- |
| Locus | Observed Heterozygosity | Expected Heterozygosity |
| PUN19 | Monomorphic | |
| PUN196 | 0.32609 | 0.35929 |
| PUN217 | 0.47826 | 0.5344 |
| PUN61 | 0.71111 | 0.67241 |
| PUN98 | 0.21739 | 0.64286 |
| STN168 | 0.32609 | 0.37912 |
| STN186 | 0.56522 | 0.5645 |
| STN217 | 0.71739 | 0.6861 |
| STN265 | 0.60465 | 0.68153 |
| STN301 | 0.80435 | 0.76613 |
| STN328 | Monomorphic | |

| **Split** | | |
| --- | --- | --- |
| Locus | Observed Heterozygosity | Expected Heterozygosity |
| PUN19 | 0.02381 | 0.02381 |
| PUN196 | 0.46809 | 0.52711 |
| PUN217 | 0.64444 | 0.66991 |
| PUN61 | 0.57447 | 0.71814 |
| PUN98 | 0.51064 | 0.52276 |
| STN168 | 0.57447 | 0.46054 |
| STN186 | 0.38298 | 0.35324 |
| STN217 | 0.67391 | 0.59795 |
| STN265 | 0.54545 | 0.58699 |
| STN301 | 0.84091 | 0.83307 |
| STN328 | Monomorphic | |

| **Stauffer** | | |
| --- | --- | --- |
| Locus | Observed Heterozygosity | Expected Heterozygosity |
| PUN19 | Monomorphic | |
| PUN196 | 0.78788 | 0.67273 |
| PUN217 | 0.68293 | 0.71635 |
| PUN61 | 0.82927 | 0.70852 |
| PUN98 | 0.35294 | 0.47366 |
| STN168 | 0.61538 | 0.5005 |
| STN186 | 0.79487 | 0.78188 |
| STN217 | 0.87179 | 0.79354 |
| STN265 | 0.75 | 0.77785 |
| STN301 | 0.91176 | 0.90737 |
| STN328 | Monomorphic | |

| **Strubel** | | |
| --- | --- | --- |
| Locus | Observed Heterozygosity | Expected Heterozygosity |
| PUN19 | 0.2439 | 0.22313 |
| PUN196 | 0.4878 | 0.48178 |
| PUN217 | 0.36585 | 0.34417 |
| PUN61 | 0.75 | 0.73481 |
| PUN98 | 0.39024 | 0.48479 |
| STN168 | 0.68293 | 0.65673 |
| STN186 | 0.82927 | 0.82686 |
| STN217 | 0.80488 | 0.82505 |
| STN265 | 0.69231 | 0.81552 |
| STN301 | 0.7 | 0.79905 |
| STN328 | 0.17073 | 0.1623 |

| **Sunglache** | | |
| --- | --- | --- |
| Locus | Observed Heterozygosity | Expected Heterozygosity |
| PUN19 | Monomorphic | |
| PUN196 | 0.5 | 0.51056 |
| PUN217 | 0.76923 | 0.72323 |
| PUN61 | 0.84615 | 0.75716 |
| PUN98 | 0.65385 | 0.52187 |
| STN168 | 0.34615 | 0.49095 |
| STN186 | 0.53846 | 0.57391 |
| STN217 | 0.61538 | 0.60709 |
| STN265 | 0.57692 | 0.57466 |
| STN301 | 0.82609 | 0.86087 |
| STN328 | 0.05556 | 0.05556 |

| **Teal** | | |
| --- | --- | --- |
| Locus | Observed Heterozygosity | Expected Heterozygosity |
| PUN19 | 0.02273 | 0.02273 |
| PUN196 | 0.40909 | 0.47623 |
| PUN217 | 0.79545 | 0.79833 |
| PUN61 | 0.68182 | 0.68809 |
| PUN98 | 0.64286 | 0.71113 |
| STN168 | 0.35556 | 0.48664 |
| STN186 | 0.71111 | 0.61623 |
| STN217 | 0.88636 | 0.86024 |
| STN265 | 0.69767 | 0.71327 |
| STN301 | 0.725 | 0.85095 |
| STN328 | 0.03704 | 0.03704 |

| **Unnamed 25** | | |
| --- | --- | --- |
| Locus | Observed Heterozygosity | Expected Heterozygosity |
| PUN19 | 0 | 0.09395 |
| PUN196 | Monomorphic | |
| PUN217 | 0.15909 | 0.18574 |
| PUN61 | 0.68182 | 0.67006 |
| PUN98 | 0 | 0.06346 |
| STN168 | Monomorphic | |
| STN186 | 0.54545 | 0.43887 |
| STN217 | 0.39535 | 0.47852 |
| STN265 | 0.69767 | 0.62408 |
| STN301 | 0.53659 | 0.55405 |
| STN328 | Monomorphic | |

| **Unnamed 30** | | |
| --- | --- | --- |
| Locus | Observed Heterozygosity | Expected Heterozygosity |
| PUN19 | 0.08333 | 0.08216 |
| PUN196 | 0.26829 | 0.23517 |
| PUN217 | 0.63415 | 0.60614 |
| PUN61 | 0.68293 | 0.69738 |
| PUN98 | 0.725 | 0.65032 |
| STN168 | 0.2 | 0.2307 |
| STN186 | 0.575 | 0.65348 |
| STN217 | 0.75 | 0.78956 |
| STN265 | 0.74286 | 0.80994 |
| STN301 | 0.92683 | 0.89852 |
| STN328 | Monomorphic | |

| **Unnamed 51** | | |
| --- | --- | --- |
| Locus | Observed Heterozygosity | Expected Heterozygosity |
| PUN19 | 0.43182 | 0.51646 |
| PUN196 | 0.47826 | 0.48829 |
| PUN217 | 0.56522 | 0.54419 |
| PUN61 | 0.65217 | 0.56713 |
| PUN98 | 0.19565 | 0.2377 |
| STN168 | 0.26087 | 0.2666 |
| STN186 | 0.67391 | 0.63617 |
| STN217 | 0.84783 | 0.81892 |
| STN265 | 0.825 | 0.83259 |
| STN301 | 0.58696 | 0.72097 |
| STN328 | 0.23077 | 0.28871 |

| **Yellowhead** | | |
| --- | --- | --- |
| Locus | Observed Heterozygosity | Expected Heterozygosity |
| PUN19 | 0.28947 | 0.25088 |
| PUN196 | 0.55 | 0.50095 |
| PUN217 | 0.275 | 0.36677 |
| PUN61 | 0.55 | 0.59272 |
| PUN98 | 0.275 | 0.36741 |
| STN168 | 0.5 | 0.4443 |
| STN186 | 0.69231 | 0.66067 |
| STN217 | 0.47368 | 0.56772 |
| STN265 | 0.71429 | 0.76936 |
| STN301 | 0.85 | 0.82184 |
| STN328 | Monomorphic | |

Table S4: Proportion of loci deviating from Hardy-Weinberg expectations when adjusted using two different corrections for multiple comparisons (Sequential Bonferroni, SB, and False Discovery Rate, FDR)

| Population | Percentage of loci deviating HWE (SB) | Percentage of loci deviating HWE (FDR) |
| --- | --- | --- |
| Adams | 9.1 | 0.0 |
| Arden | 18.2 | 18.2 |
| Beaver | 9.1 | 0.0 |
| Big North | 0.0 | 0.0 |
| Birch | 0.0 | 0.0 |
| Cowboy | 20.0 | 20.0 |
| DDTP | 11.1 | 11.1 |
| Dodds | 0.0 | 0.0 |
| Eckford | 9.1 | 0.0 |
| Fiesta | 0.0 | 0.0 |
| Frog Pond | 10.0 | 10.0 |
| Gasplant | 9.1 | 9.1 |
| Gunrange | 0.0 | 0.0 |
| Honeysuckle | 0.0 | 0.0 |
| Johnson West | 0.0 | 0.0 |
| Oz | 11.1 | 11.1 |
| Split | 0.0 | 0.0 |
| Stauffer | 0.0 | 0.0 |
| Struble | 0.0 | 0.0 |
| Sunglache | 0.0 | 0.0 |
| Teal | 0.0 | 0.0 |
| Unnamed 25 | 12.5 | 12.5 |
| Unnamed 30 | 0.0 | 0.0 |
| Unnamed 51 | 9.1 | 9.1 |
| Yellowhead | 0.0 | 0.0 |

Table S5 . The percentage of populations deviating from Hardy-Weinberg expectations (HWE) at 11 loci in brook stickleback populations in 25 small lakes in Central Alberta, when adjusted using two different corrections for multiple comparisons (Sequential Bonferroni, SB, and False Discovery Rate, FDR).

| Locus | Percentage of Populations that Deviated from HWE (SB) | Percentage of populations that deviated from HWE (FDR) |
| --- | --- | --- |
| PUN19 | 15.8 | 15.8 |
| PUN196 | 0.0 | 0.0 |
| PUN217 | 0.0 | 0.0 |
| PUN61 | 4.0 | 4.0 |
| PUN98 | 12.0 | 8.0 |
| STN168 | 4.2 | 0.0 |
| STN186 | 0.0 | 0.0 |
| STN217 | 12.0 | 12.0 |
| STN265 | 4.0 | 0.0 |
| STN301 | 4.0 | 4.0 |
| STN328 | 0.0 | 0.0 |
